# The Coexistence of Chronic Lymphocytic Leukemia and Multiple Myeloma

**DOI:** 10.4274/tjh.galenos.2018.2018.0096

**Published:** 2019-05-03

**Authors:** Ceren Hangül, Orhan Kemal Yücel, Bahar Akkaya, Levent Ündar, Sibel Berker Karaüzüm

**Affiliations:** 1 Akdeniz University Faculty of Medicine, Department of Medical Biology and Genetics, Antalya, Turkey; 2 Akdeniz University Faculty of Medicine, Department of Hematology, Antalya, Turkey; 3 Akdeniz University Faculty of Medicine, Department of Pathology, Antalya, Turkey

**Keywords:** Chronic lymphocytic leukemia, Multiple myeloma, Hematological malignancy, Clonality

## To the Editor,

Multiple myeloma (MM) and chronic lymphocytic leukemia (CLL) are neoplastic diseases originating from different stages of B-cell maturation. The coexistence of MM and CLL in the same patient is quite rare [1]. Here we report a patient with CLL who later developed kappa light chain MM.

A 67-year-old male was diagnosed with CLL with CD5+, CD19+, CD23+ B lymphocytes detected by flow cytometry. Karyotype analysis revealed 45,X,Y[4]/46,XY[10] and 55% deletion of 11q22.3 was found as the sole anomaly by fluorescence in situ hybridization (FISH). He was treated with chlorambucil and dexamethasone.

**Figure 1:**
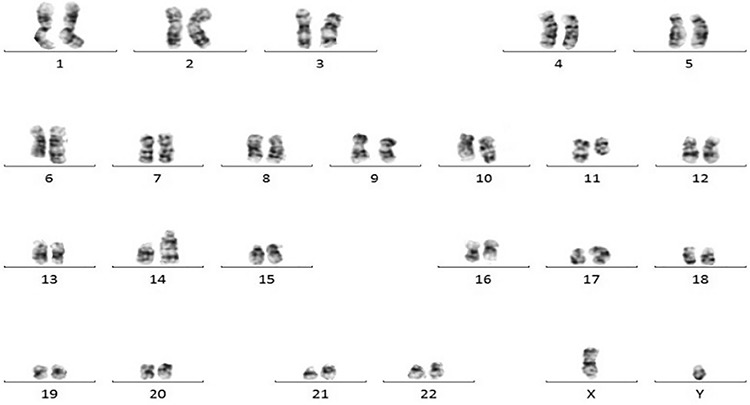
Karyotyping analysis showing 46,XY,der(6)t(1;6)(q11;q23),t(11;14)(q13;q32),dup(17)(q23q25)[17]/46,XY[2] (94x64 mm; 72x72 DPI).

Five years later, he was admitted with fatigue, back pain, hypercalcemia, and acute renal failure. There was no palpable lymphadenopathy or hepatosplenomegaly. Serum and urine immunofixation electrophoresis revealed kappa light chain monoclonal protein, while bone marrow (BM) aspiration revealed at least 30% atypical plasma cells. Similarly, BM immunophenotyping revealed 30% clonal plasma cells (CD38+, CD138+) and approximately 1% residual CLL cells. The complex karyotype was found as 46,XY,der(6)t(1;6)(q11;q23),t(11;14)(q13;q32),dup(17)(q23q25)[17]/46,XY[2] (Figure 1). FISH analysis revealed t(11;14) and deletion 6q23, but not the prior 11q22.3 deletion. He was diagnosed with MM. Intravenous hydration, plasmapheresis, furosemide, pamidronate, and dexamethasone were started. Following discharge from the hospital, he was lost to follow-up.

The coexistence of CLL and MM is quite rare and there is controversy as to whether the two diseases arise from the same clone or distinct clones. Fermand et al. [2] showed that these malignancies came from the same clone by the identification of Ig idiotypes. After CLL cells were exposed to mitogens and allogenic T cells, a class switch from IgG to IgA was observed, showing that CLL cells can be precursors of plasma cells. Brouet et al. [3], Novak et al. [4], and Kaufmann et al. [5] showed Ig molecules synthesizing different light chains, revealing the coexistence of two distinct clones. However, Ig subtyping cannot always eliminate clonality. In some cases CLL and MM are diagnosed together, whereas in others MM is diagnosed 1-15 years after CLL. Barlogie and Gale [6] indicated a difference in pathophysiology; in CLL, most tumor B cells are inert and arrested in the G0/G1 phase, whereas in MM there is an increase in proliferation with stromal cell cytokines like IL-6. In our case, there was ATM deletion at the time of the CLL diagnosis, but not at MM diagnosis, indicating multiclonality. 

Compatible with our case, there are reported cases of patients diagnosed with MM after CLL who had chromosome 11 anomalies at the time of the CLL diagnosis [7], raising the following question: Could some of the chromosome 11 anomalies be related to transformation from CLL to MM, and used as a predictor? To clarify the role of these genetic, epigenetic [8,9], or microenvironmental factors for the coexistence of two diseases, more case reports are needed.
